# JLAN: medical code prediction via joint learning attention networks and denoising mechanism

**DOI:** 10.1186/s12859-021-04520-x

**Published:** 2021-12-13

**Authors:** Xingwang Li, Yijia Zhang, Faiz ul Islam, Deshi Dong, Hao Wei, Mingyu Lu

**Affiliations:** 1 School of Information Science and Technology, Dalian Maritime University, Dalian, 116026 Liaoning China; 2 Department of Pharmacy, The First Affiliated Hospital of Dalian Medical University, Dalian, 116026 Liaoning China

**Keywords:** Automatic diagnosis, Attention mechanism, Denoising model, Joint learning, Multi-label classification

## Abstract

**Background:**

Clinical notes are documents that contain detailed information about the health status of patients. Medical codes generally accompany them. However, the manual diagnosis is costly and error-prone. Moreover, large datasets in clinical diagnosis are susceptible to noise labels because of erroneous manual annotation. Therefore, machine learning has been utilized to perform automatic diagnoses. Previous state-of-the-art (SOTA) models used convolutional neural networks to build document representations for predicting medical codes. However, the clinical notes are usually long-tailed. Moreover, most models fail to deal with the noise during code allocation. Therefore, denoising mechanism and long-tailed classification are the keys to automated coding at scale.

**Results:**

In this paper, a new joint learning model is proposed to extend our attention model for predicting medical codes from clinical notes. On the MIMIC-III-50 dataset, our model outperforms all the baselines and SOTA models in all quantitative metrics. On the MIMIC-III-full dataset, our model outperforms in the macro-F1, micro-F1, macro-AUC, and precision at eight compared to the most advanced models. In addition, after introducing the denoising mechanism, the convergence speed of the model becomes faster, and the loss of the model is reduced overall.

**Conclusions:**

The innovations of our model are threefold: firstly, the code-specific representation can be identified by adopted the self-attention mechanism and the label attention mechanism. Secondly, the performance of the long-tailed distributions can be boosted by introducing the joint learning mechanism. Thirdly, the denoising mechanism is suitable for reducing the noise effects in medical code prediction. Finally, we evaluate the effectiveness of our model on the widely-used MIMIC-III datasets and achieve new SOTA results.

## Introduction

Clinical text coding has come to the foreground in the medical field, aiming to solve the limitations of manual work. The coding system takes electronic health records (EHR) as input and outputs the prediction results of related diseases. As an essential part of EHR, clinical records contain lengthy medical history, personal details, current symptoms, and laboratory test results [1]. To avoid the repetition and ambiguity caused by the clinical texts, the World Health Organization recommends using the International Classification of Diseases (ICD) for the medical coding task.

ICD is a medical disease classification and diagnosis system. The diagnostic codes are typically accompanied by some metadata that comes from the ICD. In addition, the ICD provides an alphanumeric encoding of diagnoses and treatments, as shown in Table 1.

**Table 1:** Examples of ICD-9 codes (011-016)

ICD code	Description
011	Tuberculosis
012	respiratory tuberculosis
013	Tuberculosis of the meninges and central nervous system
014	Bowel and intestinal membrane gland tuberculosis
015	Bone and joint tuberculosis
016	Reproductive urinary system tuberculosis

The ICD coding refers to the process of assigning codes representing diagnoses and procedures. Most hospitals rely on manual coding by human coders to assign standard diagnosis codes to the discharge summaries for billing purposes. Using the ICD coding system, medical staff can quickly make clinical diagnoses of patients.

Hence, the ICD coding is aimed to assign the most probable diagnostic codes to the patients based on the clinical records. Traditionally, clinical diagnosis is made by well-trained clinical coders. However, due to the growing clinical records, manual coding has become increasingly time-wasting and error-prone. For example, in the United States, approximately 20% of patients are misdiagnosed at the primary healthcare level. Moreover, one-third of the misdiagnosis will cause serious harm to the patients sooner or later [2].

Therefore, the ICD coding task is still highly challenging. In the clinical dataset MIMIC-III [3], there is a long-tailed distribution phenomenon. More than half of the ICD codes have never appeared. In addition, ICD coding is easily affected by noise, which leads to poor prediction effects.

Specifically, there are misclassified records during code allocation, called noise samples. Recent studies [4] have shown that some neural networks may overfit noise labels and not generalize well. The samples may be noisy for multiple reasons: the ambiguity of the description, human errors, and inexperience of the annotator. While learning noise samples have been extensively studied in computer vision [5], the corresponding progress in ICD coding has been relatively limited.

**Fig. 1:**
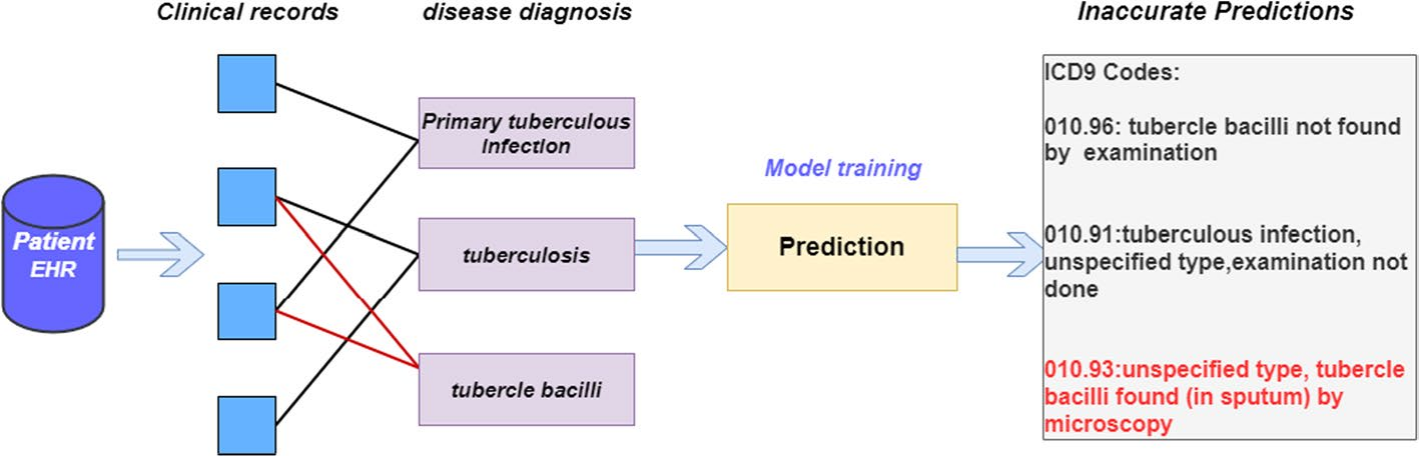
Example of noise interference

Figure 1 shows that ICD coding is affected by noise samples. As shown by the red lines, the patient's clinical records erroneously interacted with the tubercle bacilli. As a result, the patient's ICD codes were incorrectly predicted as 010.96, 010.91, and 010.93, which reduced the accuracy of code prediction. Specifically, several types of errors occur frequently [2]. Firstly, the differences between disease subtypes of the ICD codes are so subtle that it is common for coders to choose incorrect subtypes. Secondly, doctors often use abbreviations and synonyms, creating ambiguity and imprecision when coders match ICD codes to these descriptions [6]. Thirdly, there is a many-to-one relationship between the clinical texts and the ICD code in many cases. However, inexperienced coders may code for each disease separately. Moreover, the cost of coding errors and the financial investment to improve coding quality are estimated at $25 billion per year [7] in the United States. Therefore, how to utilize a denoising mechanism is particularly important.

**Fig. 2:**
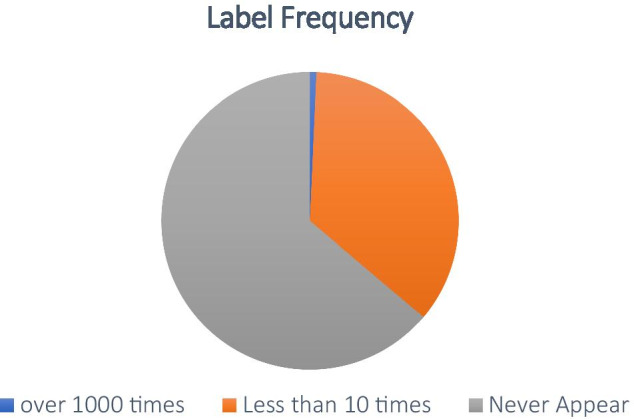
The distribution of ICD codes on MIMIC-III

In addition, the phenomenon of long-tail distribution is also a problem that ICD coding needs to solve. Specifically, a few labels have more instances, while most labels have few instances. The unbalanced number of instances brings challenges to label classification. As shown in Fig. 2, there is a long-tailed distribution in MIMIC-III. A few medical codes occur more than 1000 times; around 4000 codes arise between 1 and 10 times. Even more than 50% of medical codes have never happened. Therefore, it leads to the long-tailed distribution in the ICD coding classification [8].

Besides, electronic medical records are extremely rich in content with lengthy texts, but only part of the vital information is needed in the ICD coding process. Therefore, it is tough to find the critical data in complex EHR.

Over the past few years, some efforts have been dedicated to dealing with long-tail distribution problems. The existing methods for long-tail classification can be divided into two categories:

1) Class distribution rebalancing: Methods include under-sampling of head classes and over-sampling of tail classes [9]. Unfortunately, the rebalancing approach interferes with model performance because overemphasis on tail amplifies the impact of tail data noise [10]. In addition, the under-sampling approach makes the information learned by the model too single.

2) Another processing idea is the few-shot learning strategy: Few-shot learning [11] and long-tail classification have similar characteristics because some labels contain many instances, while others have few instances. Few-shot learning usually trains classifiers on labels with rich samples and then migrates to classes with sparse samples to improve classification performance. This approach ignores the differences between instances and leads to excessive optimization of tail classes. These methods have been applied in biomedical text mining. However, they still have large development space in handling the association between labels and texts.

In our work, we are not simply balancing data but jointly learning labels and texts to construct specific text representations for rare labels. Furthermore, the ICD coding work has also aroused research interest in academia and industry. Many machine learning and deep learning methods have been tried to solve these problems.

The supervised machine learning method trains neural networks to learn feature combinations from clinical notes in recent years. Some works also formalize multi-label classification into a ranking problem, using the ranking method to rank the categories of documents and select the corresponding labels [12].

Deep learning technology has shown substantial advantages over traditional machine learning methods and has been widely used for code allocation [13]. Most researchers model this task as a multi-label text classification problem based on EHR's free text. When solving multi-label classification problems, deep learning usually divides the problem into two parts. One is the neural document encoder, which represents documents as a continuous semantic vector [14]. The other is the prediction layer, which matches medical text space with disease code space. For example, Shi et al. [15] proposed a character-perceived Long-Short Term Memory (LSTM) network that generated written diagnosis descriptions and representations of diagnosis codes.

Moreover, some researchers incorporated external knowledge into the model. For example, Knowledge Source Integration (KSI) calculated the matching score between the clinical note and each knowledge document for this task. Baumel et al. [16] proposed a hierarchical Gate Recurrent Unit (GRU) with a label-dependent attention layer to alleviate lengthy records problems. Wang et al. [17] proposed a label-word joint embedding model and applied the cosine similarity to assign the codes.

Recently, most deep learning models see automatic diagnosis as a sequence learning problem, including the use of convolutional neural networks [18] to capture complex semantic information. On this basis, medical ontology is further introduced as auxiliary knowledge. For example, Bai et al. [19] incorporate Wikipedia into the model to enhance its predictive ability. Besides, the patient's medical history and demographic information can strengthen the prediction of future admissions.

### Our contributions

- We propose a dual attention model for ICD coding. In our model, the clinical texts related to the medical code can be identified using the self-attention and label attention mechanisms. Furthermore, the interpretability of the medical code prediction can be improved.
- We design a joint learning mechanism to effectively integrate the attention matrixes in the dual attention model to deal with long-tail distribution. In addition, we also introduce a denoising mechanism to suppress the disturbance of noise samples and accelerate the speed of model convergence.
- We evaluate our model on the MIMIC-III dataset. Experimental results show that the model obtains the new SOTA performance across evaluation metrics.

## Methods

**Fig. 3:**
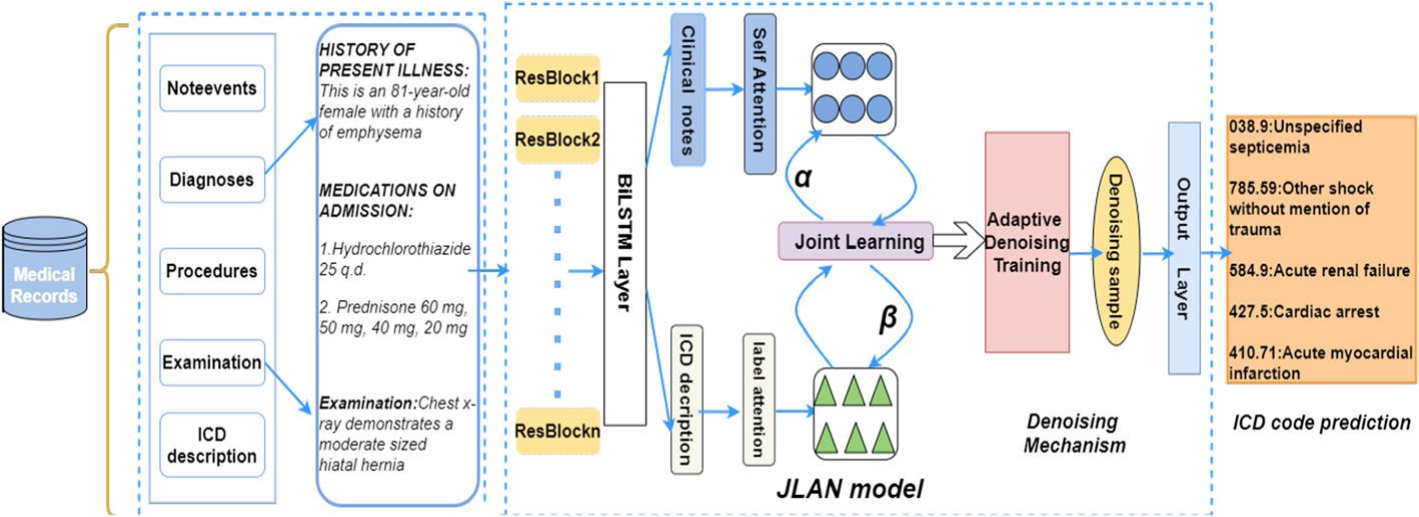
Schematic overview of JLAN

This section briefly introduces the proposed Joint Learning Attention Network (JLAN), as shown in Fig. 3.

JLAN is made up of three parts. The first part is to capture the semantic information of the dataset using a residual neural network and bidirectional long short-term memory (Bi-LSTM) network. The second part extracts appropriate information from the label attention and self-attention mechanism, called joint learning. The third part introduces a denoising mechanism to reduce the noise in the training samples and help the model converge faster. Finally, medical code prediction results have been significantly improved.

Specifically, we use the self-attention mechanism for clinical texts to identify the code-related components from each document. At the same time, we introduce the label attention mechanism to make ICD codes attend to clinical document representation. We design the joint learning strategy to output the comprehensive document representation to adapt the two parts.

In addition, we consider the noise problem of clinical diagnosis and capture the noise through an auxiliary noise model over the classifier model. We first assign a probability score to each training sample. Then, we use this score to guide the learning of the noise model selectively. Our function constrains the noise sample within the noise model and drives the classifier to learn from the clean training samples.

### Problem definition

Let ${T = \{({x}_{i}, {y}_{i})\}}_{i=1}^{N}$ denote the clinical texts, which contain N documents with related medical codes ${{Y}_{i}= \{yi\in \{\mathrm{0,1}\}}^{C}\}$. Where $C$ is the number of all labels. Every word can be encoded to a low-dimension space and represented as a $n$-dimension vector via the word2vector technique [20]. Let ${x}_{i} = \{{w}_{1},\dots \dots {w}_{n} \}$ denote the $i\_th$ clinical record, ${w}_{n}$ is the $n\_th$ word vector in the clinical record.

For the ICD coding task, each code contains text information. Therefore, the code can be represented as an embedding vector. The set of codes can be encoded by a trainable matrix $M$. Our model trains the classifier to assign the most relevant codes to the newly arriving record by learning the input document and their associated codes.

### Input representation

Word embedding has been widely used in neural networks to capture the basic semantic information of words effectively. Generally, clinical notes are written by medical professionals. Thus, we use a distributed representation to obtain a word vector closer to the meaning of the target word.

Our model uses a word list $c = \{{c}_{1}, {c}_{2}, \dots \dots , {c}_{n} \}$ as input, *n* denotes the length of the sequence. Let *E* means the word embedding matrix, which is pretrained via word2vec [20] from the dataset. Hence, the input can be replaced by a matrix $E = \{{e}_{1},{e}_{2},\dots \dots {e}_{n}\}$, ${e}_{n}$ is the word vector.

### Residual convolutional network

To solve the degradation problem of the deep neural network, we introduce the residual neural network into the model. Specifically, the residual neural network can make models converge faster and help us adopt a deeper design for the feedforward neural network. We input the word embedding matrix into the residual block [21]. Thus, the residual block can be formalized as:

1 $${Y}_{i}=F\left({E}_{i},\left\{{W}_{i}\right\}\right)+h({E}_{i})$$

2 $${E}_{i+1}=ReLU\left({Y}_{i}\right)$$

where $E,Y$ indicates the input and output of this layer, the $F({E}_{i},\{{W}_{i}\})$ indicates the residual mappings. A residual block consists of two parts. The first part goes through the convolution network and activation function, and the second part uses shortcut connections to add the input of this layer to the output of the first part. Finally, the added result is fed to the output layer through the activation function to complete the processing of residual blocks.

### Bidirectional LSTM layer

To capture each word's forward and backward contextual information in each clinical text, we adopt the Bi-LSTM model [22] to learn the word embedding of each clinical record. In addition, Bi-LSTM can keep long dependent information and overcome gradient vanishing problems. Therefore, it is fit to capture the long-term dependency feature. At time step $d$, the hidden state can be updated with the help of input and the $\left(d-1\right)\_th$ step output, we compute the vectors as:

3 $${\overrightarrow {{h_{d} }} = LSTM\left( {\overrightarrow {{h_{d - 1} }} ,w_{d} } \right)}$$

4 $${\overleftarrow {{h_{d} }} = LSTM\left( {\overleftarrow {{h_{d - 1} }} ,w_{d} } \right)}$$

5 $${h_{d} = \overrightarrow {{h_{d} }} \oplus \overleftarrow {{h_{d} }} }$$

The dimensionality of the hidden state is set to *k*, resulting in the size of Bi-LSTM vectors ${h}_{d}$ at 2*k*. Therefore, the whole document can be represented as a matrix $H=[{h}_{1},{h}_{2},\dots ,{h}_{n}]\in {R}^{2k\times n}$.

### Dual attention network

The difficulty of the long-tail problem is that most labels have rare instances. Therefore, classifying labels in a limited number of instances has become an urgent problem to be solved. The attention mechanism can give more weight to a small part of crucial information when processing extensive data. This mechanism is naturally suitable for dealing with long-tail problems. Moreover, the number of cases between different diseases varies greatly. Therefore, how to comprehensively characterize data is a challenging task. To this end, we have designed a dual attention mechanism, which can effectively link different feature information and adaptively integrate disease-related text information.

In this subsection, we introduce a dual attention network for medical code and document representation learning. This network composes of the label attention mechanism and the self-attention mechanism. We introduce these two parts in detail in the following two sub-sections.

The dual attention network aims to identify the components related to the medical code in each clinical text. Intuitively, it can simultaneously take the clinical text and medical codes into account and expand the receptive field of the model. Therefore, this strategy is suitable for clinical code classification.

For example, regarding the original text, “This is an 81-year-old woman with a history of emphysema, her primary care doctor thought she had shortness of breath for three days and thought it was a COPD attack.” It is divided into two categories: Emphysema and COPD. The content of "emphysema" is more related to the patient's medical history than directly related to symptoms, and “COPD” (chronic obstructive pulmonary disease) should be related to the patient's symptoms. Next, we introduce the two components of the dual attention network.

### Self-attention mechanism

As mentioned above, a multi-label clinical text can be marked by more than one medical code, and each clinical document should have the most relevant context to its corresponding medical code. In other words, each record may contain multiple components, which contribute differently to each medical code.

To capture the different components of each text, we adopt a self-attention mechanism [23], which has been successfully used in various text mining tasks [24]. The clinical text attention score (${T}^{S }\in {R}^{l\times n}$) can be calculated by.

6 $${T}^{S }=softmax\left({W}_{1}\mathit{tan}h\left({W}_{2}H\right)\right)$$

where ${W}_{1}\in {R}^{d\times 2k}$ and ${W}_{2}\in {R}^{l\times d}$ are the self-attention parameters that need training. The *d* is a hyperparameter that we can set. Each row ${T}_{j}^{s}$ (an *n*-dim row vector where *n* is the total number of words) represents the contribution of clinical records to the ${j}_{th}$ label. We can get the linear combination of contexts. Finally, the clinical text representation of the medical code ${M}^{(S)}\in {R}^{l\times 2k}$ is calculated as follows.

7 $${{M}_{j}^{s}=T}_{j}^{s}{H}^{T}$$

### Label attention mechanism

The self-attention mechanism can be regarded as the attention based on the clinical text because it focuses on the document content.

As we all know, medical codes have specific semantics in ICD coding. To utilize the semantic information of the codes, we preprocess the codes' descriptions and represent them as a trainable matrix ${C\in R}^{l\times k}$ in the same *k*-dim space with the documents.

Once we have the word embedding from Bi-LSTM and the code embedding in $C$, we can determine the semantic relationship between each pair of words and codes. We calculate the dot product between ${h}_{d}$ and ${C}_{j}$ as follows.

8 $${B}^{\left(l\right)}=CH$$

where ${B}^{\left(l\right)}\in$ ${R}^{l\times n}$ indicates the forward and backward sides relation between words and codes. Like the previous self-attention mechanism, the medical code representation can be constructed by linearly combining the context words of the code, as shown below.

9 $${M}^{\left(l\right)}={B}^{\left(l\right)}{H}^{T}$$

Finally, the document can be re-represented along with the code by ${M}^{\left(l\right)}\in {R}^{l\times 2k}$.

### Joint learning mechanism

Using these two pieces of information has become a vital issue when we get the label attention matrix L and the self-attention matrix S. In this section, a joint learning strategy is proposed to extract critical information from the attention matrix.

Joint learning can integrate multiple sub-models into one model. Specifically, after the label attention and self-attention matrix are determined, joint learning can train the attention modules and the rest of the model together by introducing hyperparameters. In this way, we build specific document representations for both high-frequency and low-frequency labels.

**Fig. 4:**
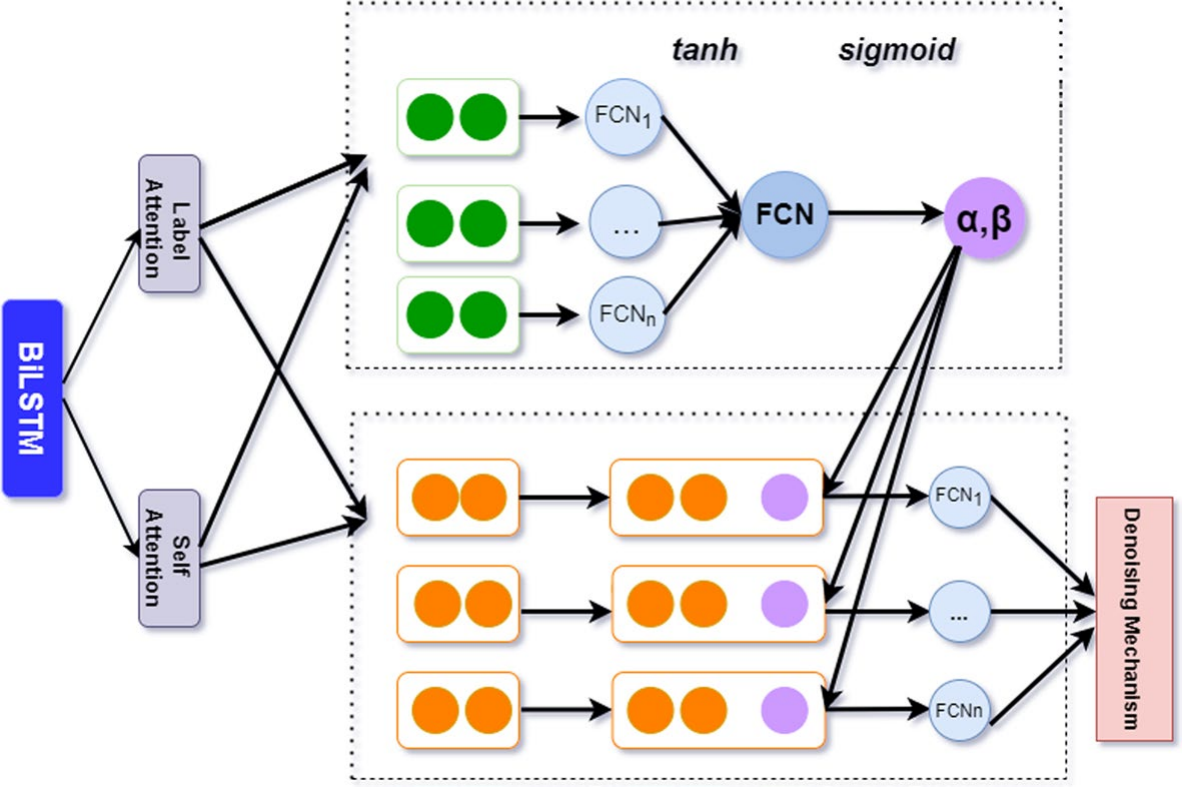
The scheme of the joint learning mechanism

The label attention matrix focuses on the semantic connection between medical code and clinical text. In contrast, the self-attention matrix focuses on the content of clinical medical records. We introduce the joint learning mechanism to fully use these two parts, as shown in Fig. 4, which can extract appropriate information from these two parts.

Specifically, we multiply the self-attention matrix and the label attention matrix with ${W}_{3} \mathrm{and} {W}_{4}$, and feed the results to the sigmoid activation function. After that, we get two weight vectors $\alpha$ and $\beta$ to represent the importance of different attention matrices. These two weight vectors can be obtained by inputting the fully connected layer on *S* and *L*.

10 $${Sigmoid \left( x \right) = \frac{1}{{1 + e^{ - x} }}}$$

11 $${\alpha = Sigmoid\left( { S W_{3} } \right), S \in R^{l \times k} }$$

12 $${\beta = Sigmoid\left( { L W_{4} } \right), L \in R^{l \times k} }$$

${W}_{3},{W}_{4}\in {R}^{k}$ are the parameters to be trained. ${\alpha }_{i}$ and ${\beta }_{i}$ represent the importance of different attention matrices to construct the final attention matrix representation for the $i\_th$ label text. Therefore, we apply the following constraints to the two weight vectors.

13 $${0 < \alpha_{{i{ }}} + \beta_{i} \le 1}$$

After that, we multiply the weight vector with the label attention and self-attention matrix. Finally, we splice the label attention matrix and the self-attention matrix after the above processing along the $i\_th$ label to obtain the attention matrix.

### Denoising mechanism

In this part, we consider the noise problem in medical code allocation. Specifically, ICD code assignment is usually a manual process that takes a long time per patient. Due to inexperienced coders, differences between coders, and incorrect grouping codes, it is also prone to errors. In addition, clinical diagnosis and treatment records are often long texts prone to misspelling or typos, leading to wrong code predictions and affect model performance[25].

Since noise negatively influences the classification results, we consider introducing the denoising mechanism and designing an auxiliary noise model on the classifier. Our target is to identify and prune the noisy samples to improve the quality of classifier training [26].

We leverage the finding that learning on clean labels is more accessible than noise labels [27]. Furthermore, we combine the binary cross entropy loss function [28] and design it as a truncation loss function. Specifically, truncation loss discards large loss samples with dynamic thresholds in each iteration. Our training goal is to minimize the loss between the prediction $\tilde{y }$ and the target y:

14 $$\begin{array}{c}{T}_{loss}\left(y,\tilde{y }\right)=\left\{\begin{array}{c}0, {BC}_{loss}(y,\tilde{y })>\varepsilon \cup (\tilde{y }=1)\\ {BC}_{loss}, Otherwise,\end{array}\right.\end{array}$$

where $\varepsilon$ denotes the pre-defined threshold and ${BC}_{loss}$ represents the binary cross entropy loss.

The truncation loss removes the noise samples whose binary cross entropy loss is larger than $\varepsilon$. Although this truncation loss is easy to explain and implement, the fixed threshold may not suit the entire training process. Because the noisy feedback typically has large loss values during the early epochs[29], the training loss value decrease as the training iterations increase. To adapt to the overall trend of training loss, we can replace the fixed threshold with a dynamic threshold function ${D}_{T}$, which changes the threshold during the training process.

15 $${D_{T} = \min \left( {\gamma T,D_{max} } \right),}$$

where $D_{max}$ is the upper bound, and $\gamma$ is a parameter to adjust the speed to achieve the maximum drop rate.

Thus, the training strategy constrains the noise and drives the classifier to learn from the clean training samples. This method can use the dynamic threshold function to truncate the loss value of the high-loss interaction to zero and discard the high-loss noise influence.

### Output layer

In this part, we feed the denoised information V into the classifier. Once we have a comprehensive representation of clinical texts and medical codes, we can build a multi-label text classifier through a multilayer perceptron with two fully connected layers. Then we use the sum-pooling operation to obtain the score $\widehat{y}$ for the ICD codes. Mathematically speaking, the predicted probability $\tilde{y }$ of each code can be estimated in the following way:

16 $$\widehat{y}=pooling\left(V\right), { \widehat{y}}_{i}=\sum_{j=1}^{n}{V}_{ij}, V\in {R}^{n\times k}$$

17 $$\tilde{y }=sigmoid\left(\widehat{y}\right)$$

Finally, the sigmoid function is used to convert the score vector into a probability vector.

## Results

In this section, we divide the results into two parts. In the first part, we introduce the dataset used in the experiments, the evaluation metrics, the setting of hyper-parameters and discussion, and the comparison between the JLAN and baseline models. In the second part, we conduct detailed ablation experiments for each component of the JLAN model, including attention mechanism, joint learning mechanism, and denoising mechanism.

### Datasets

In this paper, we conduct experiments on a real dataset: MIMIC-III ("Medical Information Mart for Intensive Care") [3], which is widely used in automatic clinical diagnosis. In addition, as shown in Table 2, we divide the dataset into the training set, validation set, and test set.

**Table 2:** Statistics of the datasets

Dataset	Vocab	Train	Valid	Test
MIMIC-III-50	59,168	8067	1574	1730
MIMIC-III	140,795	47,724	1632	3372

The dataset contains clinical data of adult patients admitted to the intensive care unit of Beth Israel Deaconess Medical Center in Boston, Massachusetts, between 2001 and 2012 to validate our method. The ICD-9 code annotated by professionals in the dataset is used as a label. We focus on discharge summary and learn the preprocessing and data separation method from Li [8].

We use the discharge summaries as the model's input for experiments. The MIMIC-III full dataset includes 8921 unique codes, 47,719, 1631, and 3372 discharge summaries used for training, validation, and testing.

The MIMIC-III top-50 setting also includes 8067, 1574, and 1730 discharge summaries used for training, validation, and testing, respectively.

### Preprocessing

Datasets are tokenized and converted to lowercase. Tokens that do not contain alphabetic characters are deleted, and tokens that appear in fewer than two training documents are replaced with a 'UNK' token. The documents are truncated to a maximum length of 2500 tokens.

### Evaluation metrics

For comprehensive comparison with previous ICD coding works, we measure the results of the JLAN model on a variety of metrics, including macro- and micro-averaged F1 and AUC (the area under the ROC curve), precision at $k(P@k\in \left\{\mathrm{5,8},15\right\})$. As detailed in Manning et al*.* [30], "micro-averaged" pooled each pair of (clinical text, medical code) sample decisions and then calculated the validity indicators of the pooled data. At the same time, the "macro-average" calculated the simple average of all codes. For example, the macro-averaged, micro-averaged precision and F1 are defined in Eqs. 18–21.

18 $$Micro-P=\frac{{\sum }_{i=1}^{I}T{P}_{i}}{{\sum }_{i=1}^{I}T{P}_{i}+F{P}_{i}}$$

19 $$Macro-P=\frac{1}{I}\sum_{i=1}^{I}\frac{T{P}_{i}}{T{P}_{i}+F{P}_{i}}$$

20 $$Micro-F=\frac{2\times \left(Micro-P\right)\times \left(Micro-R\right)}{Micro-P+Micro-R}$$

21 $$Macro-F=\frac{2\times \left(Macrp-P\right)\times \left(Macro-R\right)}{Macro-P+Macro-R}$$

### Experiment setting and hyper-parameter tuning

Our model has many hyperparameters, so it is difficult to search for the optimal value for all hyper-parameters. Therefore, some hyper-parameters are selected based on experience or previous work [18], and some hyperparameters are determined through experimental tests.

For the JLAN, the embedding size is 256, the learning rate is 0.001, the truncation loss is 0.15, and the residual block number is 1. The parameters corresponding to the weights are d = 200 for W_1_ and W_2_, k = 256 for W_3_ and W_4_.

The whole model is trained via Adam [31]. The parameters of all baselines are either adopted from their original papers or determined by experiments.

The following experiments were conducted to explore a better configuration of the truncation loss rate (T-loss) and the residual block number P of the residual convolutional layer. First, we tried different parameters for the model using MIMIC-III-full and MIMIC-III-50 datasets. The experimental results are shown in Table 3. For each setting, we evaluated five runs by randomly initializing model parameters. The results shown in the table are the average of the five runs. In addition, we empirically pre-define the in-channel and out-channel sizes of the remaining blocks.

**Table 3:** Performance comparison of using different T-loss in JLAN

config	MIMIC-III-full		MIMIC-III-50	
	Micro-F1	Macro-F1	Micro-F1	Macro-F1
T-loss=0.05	0.542	0.061	0.623	0.571
T-loss=0.1	0.557	0.068	0.626	0.574
T-loss=0.15	0.556	0.068	0.627	0.573
T-loss=0.2	0.547	0.064	0.625	0.573

As shown in Table 3, during the initial increase in truncation loss, performance improves in both the MIMIC-III-full and the MIMIC-III-50 settings. When the truncation loss increases to 0.1–0.15, the performance reaches the peak. However, as the truncation loss continues to increase, the model performance begins to decline. After exhaustive comparisons, we finally set T-loss to 0.15.

In addition, as shown in Table 4, the performance deteriorates as the number of residual blocks increases. The model performs best when the residual block number is 1. Therefore, we apply the optimal configuration of the residual block and the truncation loss to JLAN. Experimental results show that the performance of the combined model is further improved. Therefore, we retained this configuration in other experiments.

**Table 4:** Performance comparison of using different residual blocks in JLAN

Config	MIMIC-III-full		MIMIC-III-50	
	Micro-F1	Macro-F1	Micro-F1	Macro-F1
P = 1	0.543	0.062	0.637	0.585
P = 2	0.541	0.059	0.597	0.558
P = 3	0.540	0.059	0.582	0.524

### Baseline models

- CNN: One-dimensional convolutional neural network [32] was adopted by Mullenbach et al. for ICD coding tasks on MIMIC datasets.
- LR: Logistic Regression built a binary one-to-many classifier by training all the labels in the dataset and explored the ICD coding task on the MIMIC dataset [18].
- CAML: CNN with a label-wise attention mechanism was proposed by Mullenbach [18]. This model performs well on the MIMIC-III dataset, which contains the CNN layer and attention layer to process clinical text and medical code, respectively.
- DR-CAML: Description Regularized CAML is an extension of the CAML model, incorporating the text description of each medical code to regularize the model.
- MSATT-KG: The model consists of densely connected convolutional neural networks that produce variable n-gram characteristics and multi-scale feature attention. In this model [33], a graph convolutional neural network [34] was also used to capture hierarchical relationships between medical texts and codes.
- Bi-GRU: Bi-directional Gated Recurrent Unit [35] was used for multi-label classification. The document representation is set as the last concatenated hidden state h(t) to finish the coding task.
- LEAM: The model is proposed for the text classification task by projecting labels and words in the same embedding space and using the cosine similarity to predict the label [17].
- MultiResCNN: The Multi-Filter Residual Convolutional Neural Network was proposed by Li [8] for ICD coding. This model achieved SOTA results on the MIMIC-III dataset, utilizing multi-filter convolutional neural networks and residual networks for automatic diagnosis. In addition, it integrates label attention to enrich the semantic knowledge of the model. Therefore, this model does an excellent job of coding.

### Comparison with baseline models

#### MIMIC-III-50

**Fig. 5:**
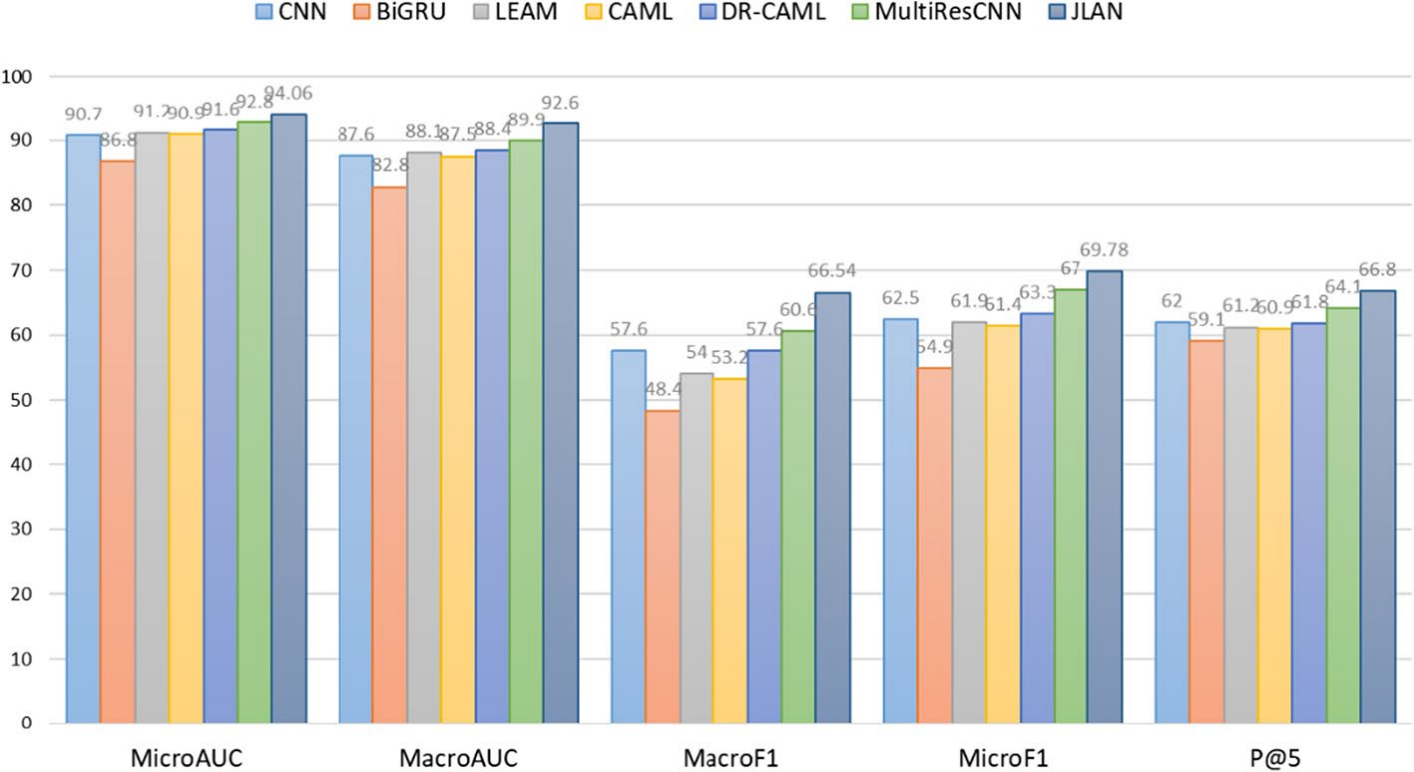
Comparison of JLAN and baseline model

Table 5 and Fig. 5 shows experimental results on the MIMIC-III-50 dataset. JLAN outperforms all the baseline models across all evaluation metrics. Compared with the SOTA model, our model improves the macro-F1, micro-F1, macro-AUC, micro-AUC, P@5 by 4.2%, 1.9%, 1.3%, 0.5%, 4.2%, respectively.

**Table 5:** The performance of the JLAN model and baseline models on the MIMIC-III-50 test set

Model	AUC		F1		P@5	R@5
	Macro	Micro	Macro	Micro		
CNN	87.6	90.7	57.6	62.5	62.0	–
BiGRU	82.8	86.8	48.4	54.9	59.1	–
LEAM	88.1	91.2	54.0	61.9	61.2	–
CAML	87.5	90.9	53.2	61.4	60.9	–
DR–CAML	88.4	91.6	57.6	63.3	61.8	–
MSATT-KG	91.4	93.6	63.8	68.4	64.4	-
MultiResCNN	89.9	92.8	60.6	67.0	64.1	62.1
JLAN	**92.6**	**94.1**	**66.5**	**69.7**	**66.8**	**63.8**

Compared with MIMIC-III-full experiments, joint learning performs better on small sample learning, which helps us transfer this training method to other tasks.

#### MIMIC-III-full

On the MIMIC-III-full dataset, Table 6 shows the evaluation results of all quantitative indicators. Specifically, using the attention mechanism (CAML and MutiResCNN) produces better performance than both traditional machine learning (LR) and deep learning models (CNN and BiGRU). Our model achieves better results in the macro-AUC, macro-F1, micro-F1, precision@5, and precision@8 than MSATT-KG and MultiResCNN, producing a slightly lower micro-AUC and P@15 than that of MSATT-KG and MultiResCNN. Specifically, our model improved the macro-F1, micro-F1, macro-AUC, P@8 by 7.8%, 2.5%, 0.88%, 0.95%, respectively.

**Table 6:** The performance of JLAN and the baseline models on the MIMIC-III-full test set

Model	AUC		F1		P@15	P@8
	Macro	Micro	Macro	Micro		
LR	56.1	93.7	1.1	27.2	–	54.2
CNN	80.6	96.9	4.2	41.9	–	58.1
BiGRU	82.2	97.1	3.8	41.7		58.5
CAML	89.5	98.6	8.8	53.9	–	70.9
DR-CAML	89.7	98.5	8.6	52.9	–	69.0
MSATT-KG	91.0	**99.2**	9.0	55.3	–	72.8
MultiResCNN	91.0	98.6	8.5	55.2	**58.4**	73.4
JLAN	**91.8**	98.8	**9.7**	**56.7**	57.9	**74.1**

Since the macro metrics focus on evaluating rare-label allocation performance, the JLAN model is better in dealing with long-tail distribution and is more suitable for dealing with this kind of problem.

### Ablation study

In this section, we evaluate the role of each component in the JLAN model. We set the following three groups of experiments to test the contribution of attention mechanism, joint learning strategy, and denoising mechanism to the model.

#### Effect of the attention mechanism

**Fig. 6:**
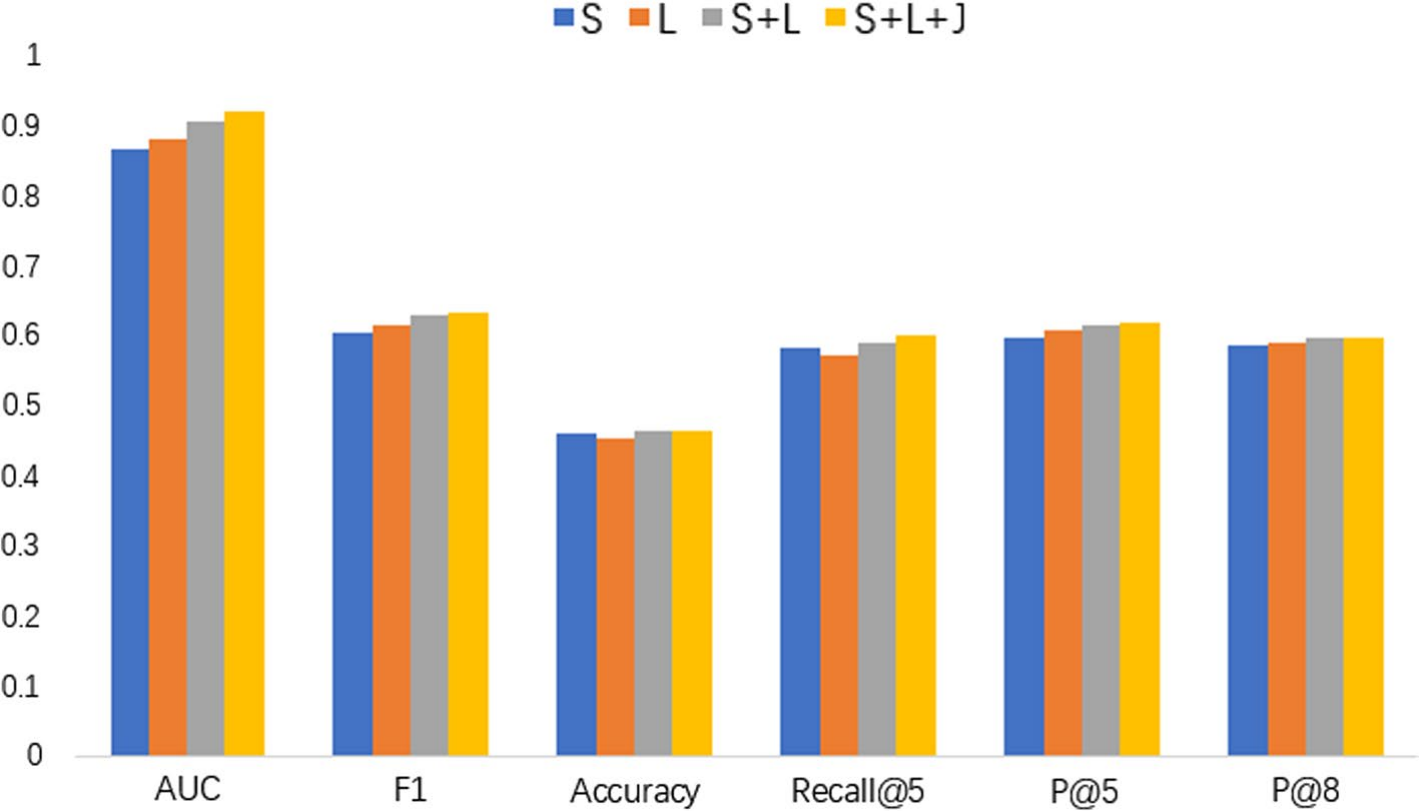
Result of the ablation experiment. 'L', 'S' and 'J' denote label attention, self-attention and joint learning, respectively

Figure 6 lists the prediction results of the MIMIC-III dataset in the form of AUC, F_1_, Accuracy, P@5, P@8, and Recall@5. L, S, and J denote the label attention, self-attention, and joint learning, respectively. As we can see, the model that uses the label attention or self-attention only performed the worst. In contrast, the model that used the above part can do better. Specifically, with the addition of model components, the model's performance improves, proving the model's effectiveness in this paper.

As for the document representation of medical codes, self-attention prefers to look for the patient's clinical records, but it ignores the information of the medical codes. On the other, label attention utilizes the advantages of the medical codes to determine the semantic relationship between the clinical texts and the medical codes. However, the medical codes do not easily distinguish the differences (e.g., combining systolic heart failure and diastolic heart failure), so it is reasonable to consider both records and codes. Therefore, we propose a joint learning mechanism. In addition, the adaptive extraction of appropriate information from these two points of concern facilitates the ICD coding task. To further verify the effectiveness of joint learning, we evaluate the joint learning mechanism separately in the next section.

#### Effect of the Joint learning

To test the importance of joint learning in the training process, we test the model's performance with and without joint learning on MIMIC-III top-50. Specifically, we intercept the model's performance over the first 50 rounds, use F1, AUC, and P@5 metrics to measure it.

For joint learning, it is difficult to compare it fairly with another model. Therefore, we design a new model that does not use joint learning. We still introduce the self-attention and label attention parts to this model and add them together, rather than training their weights.

**Fig. 7:**
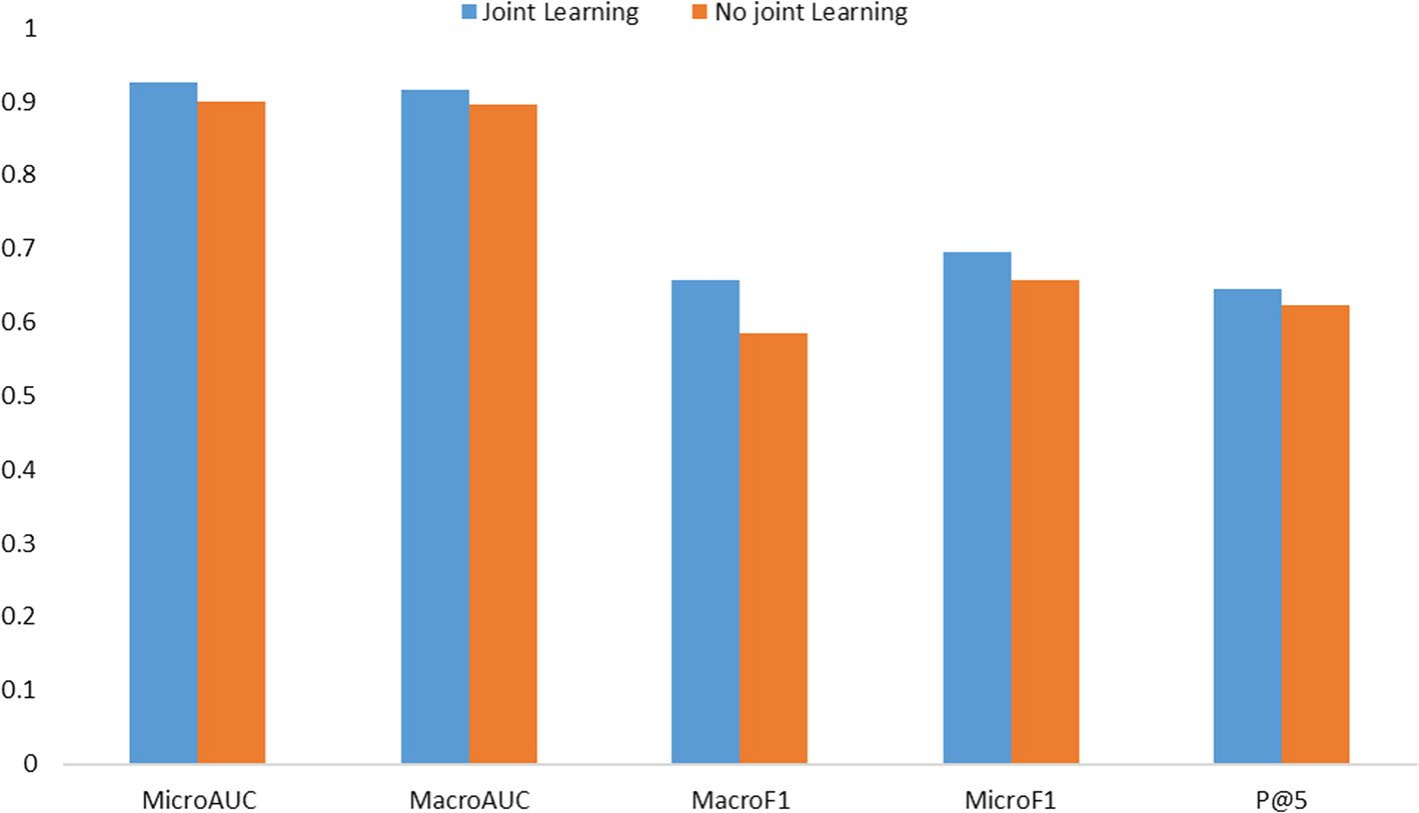
Results of the joint learning experiment. The blue and orange rectangles represent training with and without the joint learning mechanism, respectively

As Fig. 7 shows, the model using joint learning performs better overall. Specifically, we analyze that if joint learning is not used, helpful information cannot be selected adaptively even if the self-attention matrix and label attention matrix are generated. On the contrary, after introducing joint learning, the model can train the correlation coefficients for the two matrices respectively and integrate the information of the two matrices. The experimental results suggest the joint learning can effectively improve the performance of medical code prediction.

#### Effect of the denoising mechanism

This part analyzes how the denoising mechanism affects the model's performance. We choose two groups of experiments whether the denoising mechanism is used as a comparative experiment.

**Fig. 8:**
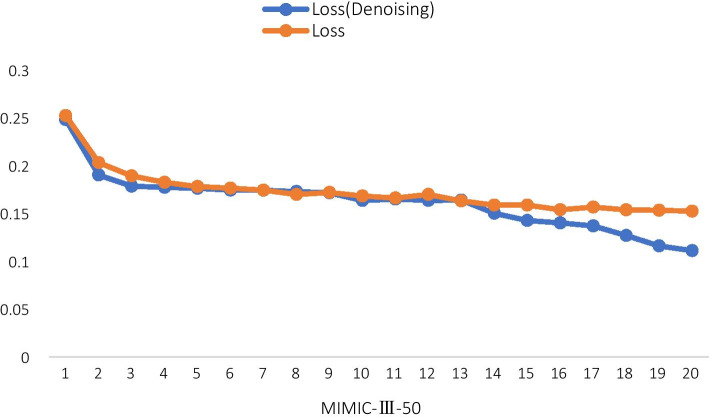
Effect of the denoising model

As Fig. 8 shows, the denoising model performs better most of the time and has less loss during training. Furthermore, the loss of the denoising model decreases faster, which is conducive to the rapid convergence of the model. By analyzing the above experimental results, we believe that by introducing the denoising mechanism, the model can quickly learn from clean samples at the early stage of training, shorten the training cycle, and thus have less loss and faster convergence. The results also prove the effectiveness of the denoising mechanism.

As the model iteration reaches our default value, the classifier no longer drops the samples but learns further from the remaining samples. This approach can ensure the integrity of dataset information and prevent the problem of over-fitting the model.

## Discussion

There is a growing demand to interpret model predictions in ways that humans can understand for predictive applications such as medical diagnosis. Although automated models are set up to reduce human error, observing which parts of labels and text contribute to the prediction improves the reliability and transparency of the model. In this section, we mainly discuss two things. Firstly, we visualize the self-attention and label attention mechanism of the model. Secondly, we discuss the limitations of this work.

First, we elect part of the clinical records of one patient, whom we call patient-A. Considering the privacy issues, we remove personal information. Second, we visualize the clinical records of patient-A using Word-Cloud; the size of the words represents the frequency of the phrase in the text, the shade of the color represents the attention weight.

**Fig. 9:**
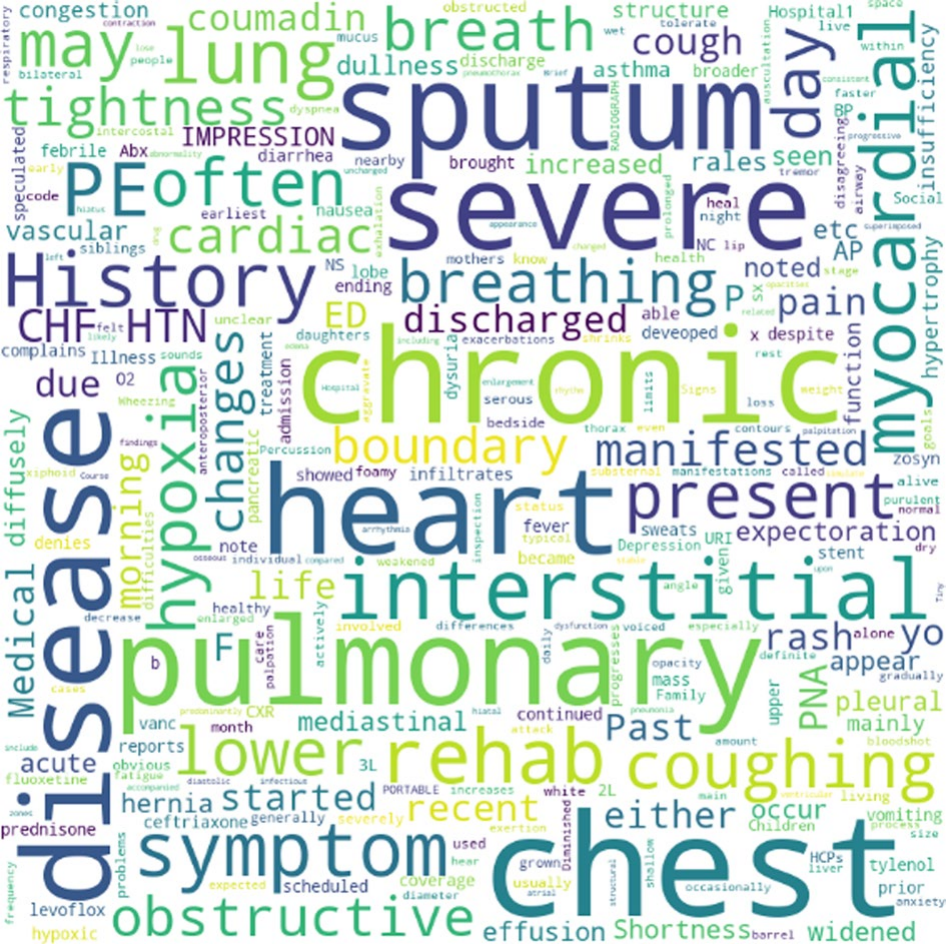
Visualization of self-attention mechanism on patient-A

As shown in Fig. 9, the self-attention mechanism pays attention to some representative words, such as "pulmonary, heart, chronic." From this, we may speculate that patient-A suffers from heart and lung diseases, verified by the information highlighted in the figure. In order to verify this conjecture, we also visualized the description of the ICD code assigned to patient-A, which is part of label attention processing.

**Fig. 10:**
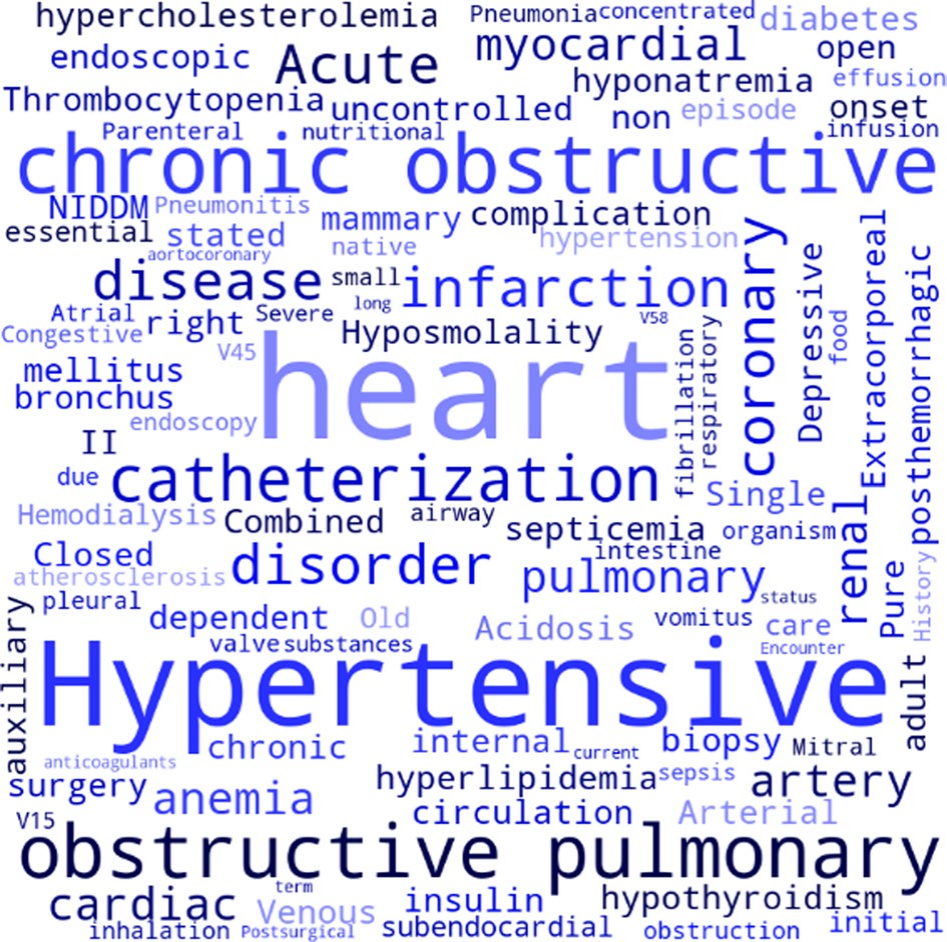
Visualization of label attention mechanism on patient-A

We can observe from Fig. 10 that patient-A suffers from chronic obstructive pulmonary disease, hypertensive heart disease, and other diseases, which confirms the previous speculation to a certain extent. Therefore, the clinical records can be matched with the medical codes by extracting critical information. The attention mechanism can assign greater weight to vital information. Through this weight allocation strategy, the JLAN model can do better in the long tail problem.

In addition, a patient may have multiple diseases, which means that the patient has several different ICD codes. Therefore, the JLAN model can highlight different essential information for different disease codes of the patient, which also provides interpretability for the model.

### Limitations

In this paper, improved performance mainly comes from three aspects: attention mechanism, joint learning strategy, and denoising mechanism. Transformer-based architectures have become the most advanced technology in almost all Natural Language Processing fields due to their ability to handle long-distance dependencies. In the future, we will explore how to introduce bidirectional encoder representations from transformers (BERT) [36] into ICD coding tasks. It is well known that BERT [37] specifies a maximum input length and requires many computational resources. Therefore, we plan to introduce sliding windows to segment clinical texts to solve the limitation of input length or introduce a self-distillation mechanism [38] to BERT.

Due to the limitation of computing resources, we do not use a larger dataset in this study. We plan to introduce larger-scale database resources and multi-modal datasets, such as "MedPix" and "Musculoskeletal Radiographs (MURA)," in the future. We will further explore ICD coding work on large-scale datasets. All in all, these are subject to further research and experiments in the future.

## Conclusions

In this paper, we proposed a joint learning attention network for ICD coding. We introduced the denoising mechanism to assist the classifier in reducing noise sample impacts during training. The experimental results on the MIMIC-III dataset showed that our model achieved the most advanced performance in various evaluation metrics. In addition, the ablation experiments proved that the denoising training strategy could effectively reduce the interference of noise and help the model converge quickly. The joint learning mechanism also improved the performances for long-tailed distribution, resulting in higher macro-averaged metrics. Our model can deal with ICD coding and be extended to be a baseline for other text classification tasks.

## Data Availability

The MIMIC-III dataset is available at https://physionet.org/content.

## Abbreviations

Bi-LSTM: Bidirectional long short-term memory
Bi-GRU: Bi-directional gated recurrent unit
BERT: Bidirectional encoder representations from transformers
COPD: Chronic obstructive pulmonary disease
CNN: Convolutional neural network
EHR: Electronic health records
GRU: Gated recurrent unit
ICD: International Classification of Diseases
KSI: Knowledge source integration
LSTM: Long short-term memory
SOTA: State-of-the-art
T-loss: Truncation loss
